# Totally embedded hybrid thin films of carbon nanotubes and silver nanowires as flat homogenous flexible transparent conductors

**DOI:** 10.1038/srep38453

**Published:** 2016-12-08

**Authors:** Suresh Kumar Raman Pillai, Jing Wang, Yilei Wang, Md Moniruzzaman Sk, Ari Bimo Prakoso, Rusli, Mary B. Chan-Park

**Affiliations:** 1 School of Chemical and Biomedical Engineering, Nanyang Technological University, 62 Nanyang Drive, Singapore 637459, Singapore; 2 School of Electrical and Electronics Engineering, Nanyang Technological University, 50 Nanyang Avenue, Singapore 639798, Singapore

## Abstract

There is a great need for viable alternatives to today’s transparent conductive film using largely indium tin oxide. We report the fabrication of a new type of flexible transparent conductive film using silver nanowires (AgNW) and single-walled carbon nanotube (SWCNT) networks which are fully embedded in a UV curable resin substrate. The hybrid SWCNTs-AgNWs film is relatively flat so that the RMS roughness of the top surface of the film is 3 nm. Addition of SWCNTs networks make the film resistance uniform; without SWCNTs, sheet resistance of the surface composed of just AgNWs in resin varies from 20 Ω/sq to 10^7 ^Ω/sq. With addition of SWCNTs embedded in the resin, sheet resistance of the hybrid film is 29 ± 5 Ω/sq and uniform across the 47 mm diameter film discs; further, the optimized film has 85% transparency. Our lamination-transfer UV process doesn’t need solvent for sacrificial substrate removal and leads to good mechanical interlocking of the nano-material networks. Additionally, electrochemical study of the film for supercapacitors application showed an impressive 10 times higher current in cyclic voltammograms compared to the control without SWCNTs. Our fabrication method is simple, cost effective and enables the large-scale fabrication of flat and flexible transparent conductive films.

Transparent conductive films are important components in numerous applications such as solar cells, display technologies, and lighting^1^,^2^,^3^,^4^,^5^,^6^,^7^. Indium Tin Oxide (ITO), which has high transparency and low sheet resistance, is currently the most commonly used transparent conductor material^8^. ITO thin films with sheet resistance as low as 10 Ω/sq and high (85%) optical transmittance at 550 nm wavelength are commercially available. However, ITO film is inherently brittle, resulting in deterioration of electronic properties when bent and this deficiency pampers its integration into flexible devices^9^. Also the high price and scarcity of Indium limits its large-scale application in less costly devices^10^; ITO is normally deposited on substrates by sputtering, and the process is batch-wise and expensive. Various other materials based on conducting polymers^11^, metal nanowires^12^,^13^, conductive oxides^14^ and carbon nanomaterials^1^ have been investigated as alternative replacement transparent flexible conductor for ITO. Conducting polymers have good electrical, mechanical and optical properties, but are highly sensitive to environmental conditions such as humidity and temperature which degrade their electrical conductivity^15^. Among these alternatives, metal nanowire-based films can reach sheet resistance of less than 10 Ω/sq at 90% transmission^16^.

Though AgNW films deposited on substrates by various methods such as spray coating^17^, vacuum filtration^18^, Mayer rod coating^19^, and spin coating^20^ show electrical and optical properties comparable to ITO films, these films still suffer from some limitations when applied to real devices. The adhesion of AgNW films deposited on substrates by these various methods is insufficient to hold the film to the substrate over the life of the device. Further, most present AgNW films consist of irregular aggregations of AgNWs protruding out (>100 nm) from the surface. For some transparent conductor applications as in solar cell devices, the device gap is only a few hundred nanometers. Since the thin film devices have gaps that are normally ~100 nm in thickness, protruding AgNWs provide pathways for electrical shorts and hence such films are unsuitable for use as thin-film electrodes^21^. Hence, the conductor surface must have roughness of about 1 nm or less. However, the diameters of metal nanowires are usually a few hundred nanometers so that they need to flattened or embedded. Further, the holes in between the nanowires need to be conductive too so that current will be homogeneous.

Embedding AgNWs into polymer film is a promising way to improve the adhesion and reduce the height variation of the surface. AgNWs have been embedded into polymeric substrates such as polyvinyl alcohol (PVA)^22^, cross-linked polyacrylates^23^,^24^,^25^ and polyurethane optical adhesive^21^. Though these methods produce AgNW composites with conductivity and transparency comparable to ITO, non-conductive voids between the nanowires remain an issue for many applications such as flexible OLEDs and photovoltaic (OPV) devices^26^,^27^. Incorporation of conductive nano-materials such as carbon nanotubes or graphene to fill the voids between the nanowire networks could improve the conductance coverage of the resulting hybrid films. Films based on carbon nano-materials such as single-walled carbon nanotubes (SWCNTs) and graphene have been of interest due to their good electrical, mechanical, optical properties and chemical stability^28^,^29^. However, the fabrication of fully embedded SWCNT-nanowire hybrid films which are flat and transparent and conductive have not been reported.

Hybrid films are of interest since they combine the desirable attributes of the component materials. Hybrid transparent conductors that integrate thin films of metal nanowires and graphene film have recently been reported^27^,^30^,^31^,^32^,^33^,^34^,^35^. Kholmanov *et al*.^33^ reported hybrid conductive electrodes by solution process using reduced graphene oxide and copper nanowires with sheet resistance of 34 Ω/sq and 80% transparency. Hybrid films of CVD grown graphene-copper grid have achieved sheet resistance of 3 Ω/sq with 80% optical transparency^30^. Scaling up of this hybrid film is difficult, requiring fabrication of large area of single- or few-layer graphene film. CVD production of graphene film for large scale applications is expensive. Another problem with CVD deposited graphene film is the complexity of the process for transfer of graphene film from the copper foil to the desired substrate. SWCNT thin films offer the advantages of high conductivity, transparency and solution-processibility. However, like metal nanowire thin films, they usually needed to have better mechanical integrity and the nanotubes need to be interlocked.

In this work, we demonstrate a flexible, flat and homogeneous transparent conductive film composed from a hybrid network comprising silver nanowires and single-walled carbon nanotubes (SWCNTs), both of which are fully embedded within a UV cured resin (Fig. 1). These hybrid polyester (PET)-based flexible films show sheet resistance of 29 ± 5 Ω/sq and 85% transparency at λ = 550 nm. The embedded hybrid SWCNT-AgNW film shows consistent resistance across multiple points on the entire surface, unlike the AgNW thin film (control) which shows no conductivity in the voids between the nanowires. Our process steps involve transfer printing and do not involve any solvent. Since the silver nanowires are embedded in a resin environment, the adhesion of the nanowires to the substrate is good and the nanowires does not easily detach from the substrate as in the case of nanowires that is loosely deposited on the PET substrate. Further, both the UV cured resin and the SWCNT thin film act as a sacrificial protective layer for the AgNW film and prevents oxidation of the silver nanowires. The potential application of the hybrid film as a supercapacitor is also demonstrated.

## Results

**Figure 1:**
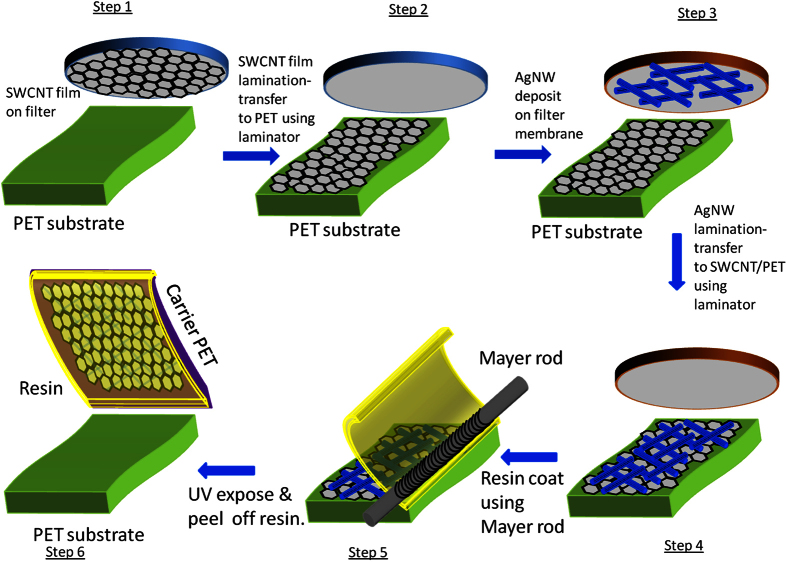
Schematic diagram of the fabrication procedure for flat embedded SWCNT-AgNW hybrid transparent conductive film on UV cured resin. Step 1: SWCNTs are deposited on a cellulose acetate filter membrane; Step 2: The SWCNT film from the filter membrane is transferred onto a PET substrate; Step 3: AgNWs are deposited on cellulose acetate membrane; Step 4: The silver nanowire film from the filter membrane is transferred onto the previously prepared SWCNT film on PET substrate; Step 5: UV resin is coated on top of the SWCNT-AgNW thin film; Step 6: Another PET carrier is placed on top of the UV resin and then cured by exposure to UV lamp. The fabricated film is then peeled off from the original PET substrate.

**Figure 2:**
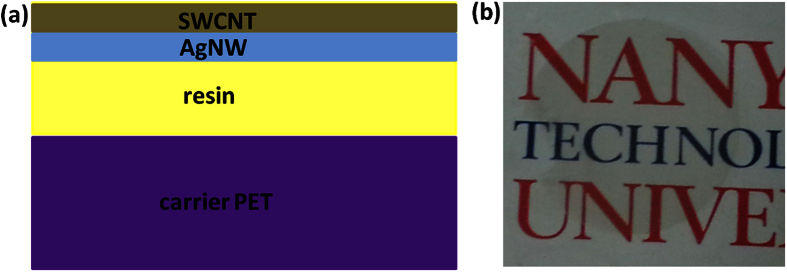
(**a**) Schematic cross-section of SWCNT-AgNW-resin-PET hybrid film, (**b**) Photographic image of the hybrid film.

Figure 1 schematically illustrates the fabrication process for our SWCNT-AgNW-resin-PET hybrid film using all-solution-based transfer processes involving silver nanowires (AgNW) and SWCNTs network embedded with a UV cured thin film atop a PET carrier (as described in the Experimental Methods). In Step 1, the SWCNT dispersion is deposited on a filter membrane to produce a uniform thin film that is transparent. In Step 2 (Fig. 1), the SWCNT thin film is transferred from the filter membrane to a PET (temporary) substrate using a laminator operated at 85 °C. The laminating transfer process does not involve any solvent treatment (such as with acetone^36^) otherwise needed to dissolve the cellulose acetate filter membrane after the transfer process. In Step 3 (Fig. 1), vacuum filtration is also applied for preparing the AgNW thin film on a filter membrane. In Step 4 (Fig. 1), the AgNW thin film is transfer-printed onto the PET substrate. In Step 5 (Fig. 1), a UV resin formulation (without any solvent) was Mayer rod-coated on top of the SWCNT-AgNW thin film. In Step 6 (Fig. 1), another PET carrier was gently placed on top of the UV resin which was then cured by exposure to a UV lamp; the entire SWCNT-AgNW-resin-PET composite hybrid film (Fig. 2a) was then lifted-off from the PET substrate.

Figure 2a shows the schematic of the cross-section of the hybrid film. The SWCNT-AgNWs that were at the bottom of the composite film in contact with the original PET substrate became the outer surface of the composite film when released from the PET substrate. Figure 2b shows the photographic image of a hybrid film. The P2-SWCNTs used were characterized by solution UV-vis-NIR spectroscopy, confirming the quality of the nanotubes (Figure S1). The SWCNT-AgNWs were glued together by the UV resin. Raman spectroscopy of the SWCNT-AgNW-resin-PET hybrid film was performed using a Renishaw Raman microscope with 633 nm laser wavelength and is shown in Figure S2. The controls used were an AgNW-resin-PET hybrid film and PET itself. The SWCNT-AgNW hybrid film on resin shows two Raman G bands (G^+^ and G^−^) at around 1615 cm^−1^ and 1592 cm^−1^ confirming the presence of SWCNTs; and they are due to atomic displacements along the tube axis (longitudinal) and radial directions of the carbon nanotube^37^. The above characteristic peaks of SWCNTs are absent for the spectra of resin with transfer of only AgNW film, and that of PET alone. Other major peaks at 1094 cm^−1^, 1294 cm^−1^, 1615 cm^−1^ and 1727 cm^−1^ are from PET substrate. The Raman spectra results corroborate the transfer of SWCNTs into the hybrid film.

**Figure 3:**
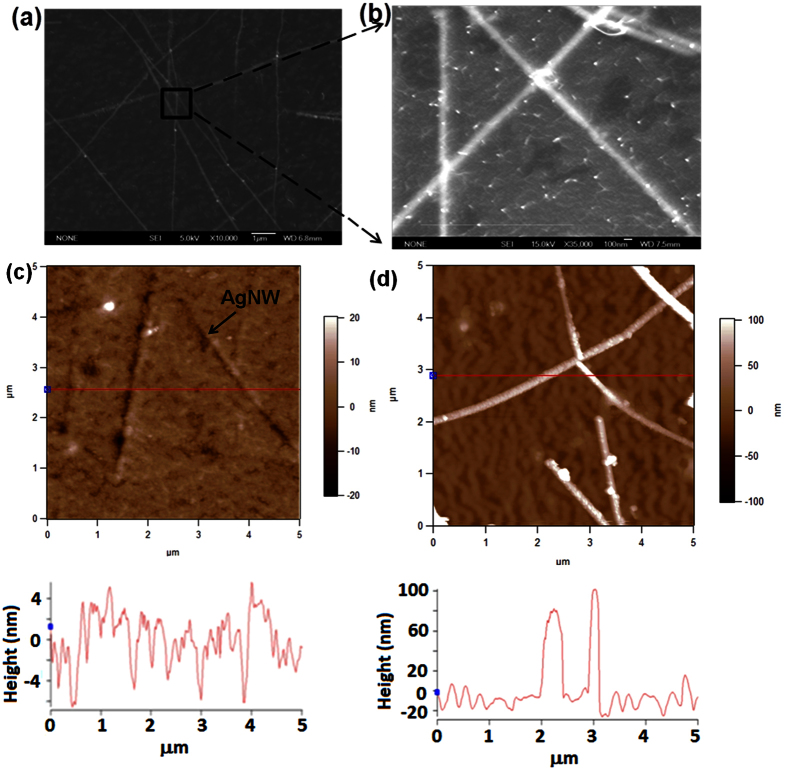
FE-SEM images of the surface of the SWCNT-AgNW-resin-PET hybrid film (**a**) 10K and (**b**) 35 K magnifications. AFM image of (**c**) SWCNT-AgNW hybrid film transferred onto UV cured resin. Average surface roughness (rms) of SWCNT-AgNW-resin-PET film is 3 nm. (**d**) silver nanowire on PET substrate.

Figure 3(a & b) shows the FE-SEM images of the surfaces of the SWCNT-AgNW-resin-PET hybrid film. The AgNWs with diameter ~115 nm are randomly oriented and the AgNW network appears to be buried under SWCNT-resin film. The transfer of AgNWs from the filter membrane appears uniform over the entire area of the film leading to uniform density of nanowires everywhere on the substrate. Figure 3b shows that the transfer of SWCNTs is also uniform over the entire area of the film, and fills the voids between the crisscross of the nanowires and they are also embedded under the resin. The surface topography of SWCNT-AgNW-resin-PET film appears to be smooth with the AgNWs and SWCNTs embedded beneath. This was confirmed by AFM imaging of the final hybrid film. AFM spectroscopy (Fig. 3c) shows that the height undulation of the hybrid film is significantly reduced to <10 nm which is significantly smaller than the AgNW diameter which is ~100 nm. (Fig. 3d shows that the diameter of the AgNWs solution-cast on a PET (without the transfer process) to be about 100 nm.) Also, the AFM image in Fig. 3c clearly shows that the AgNWs are embedded within the cured resin matrix. The surface of the film areas that are not occupied by the AgNWs are composed of SWCNT and the resin matrix. We also compared the sheet resistance of the “rough” SWCNT-AgNW hybrid on the PET before transfer (Step 4, Fig. 1) with that of the “smooth” final transferred SWCNT-AgNW-resin-PET hybrid film (Step 6, Fig. 1) and the 2 values are almost the same; for example for formulation 3 (Table 1), the values are 27 ± 6 Ω/sq versus 29 ± 5 Ω/sq, respectively. This indicates complete transfer of AgNWs and SWCNTs onto the cured resin as well as the excellent mechanical interlocking of each of the two networks of AgNWs and SWCNTs. Our lamination-transfer process of the networks forces the nanowires and nanotubes with large surface area to be compressed together and the crisscross points between these nano-materials to be tight so inter-tube/wire junction resistance appears to be minimum. The lamination process of transferring AgNW film onto the SWCNT film also improves the electrical contact between AgNW and SWCNT network films (Step 4, Fig. 1). This avoids the heating process that is typically employed by others to improve the contact of such hybrid networks^38^,^39^,^40^. Our hybrid film’s resistance is 29 Ω/sq compared to 132 Ω/sq reported by Tokuno *et al*.^41^. On the other hand, the resin appears to completely encapsulate the SWCNT-AgNW network. The liquid resin is able to penetrate into the SWCNT-AgNW hybrid network due to its low viscosity and surface energy. Our resin is formulated with low viscosity and also a surface tension modifier (see discussions below).

**Figure 4:**
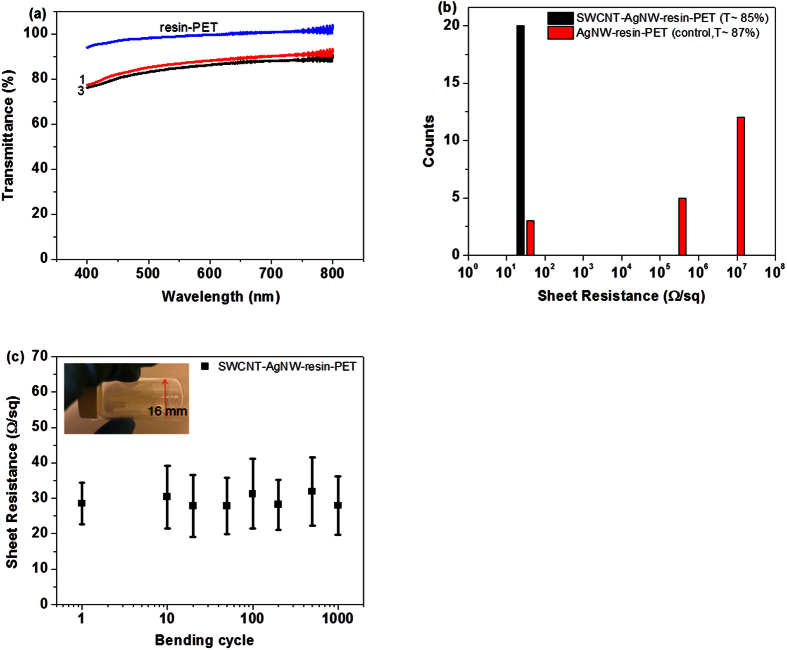
(**a**) Transmittance of resin-PET film, AgNW-resin-PET film (Table 1, #1) and SWCNT-AgNW-resin-PET film (Table 1, #3); (**b**) Sheet resistance of AgNW-resin-PET film (#1) and SWCNT-AgNW-resin-PET film (#3). 20 points were measured for each sample; (**c**) Flexibility test of SWCNT-AgNW-resin-PET film. The plot shows sheet resistance of SWCNT-AgNW-resin-PET film as a function of bending cycles, upto 1000 cycles. Inset shows the extent to which the SWCNT-AgNW-resin-PET film was bent in the flexibility test.

Figure 4(a) shows the transmittance of the optimized AgNW-resin-PET film and SWCNT-AgNW-resin-PET film; the volume of AgNW suspension (0.5 wt%) used on both films is 20 μL/47 mm diameter film and the amount of SWCNT suspension (0.2 mg/ml) added is 0.1 mL/47 mm diameter film (Table 1, formulation 3). The optical transmittance at 550 nm for the control (AgNW-resin-PET) is ~87% which decreases slightly to 85% for SWCNT-AgNW-resin-PET hybrid film. The optical transmittance of the neat UV cured resin film on PET is 99% (Fig. 4a), confirming that our resin is highly transparent. However, we need to add the other nano-materials to endow the hybrid film with conductivity.

We compared the sheet resistance and transmittance performance of our hybrid film with other hybrid transparent conductors reported in literature using solution processed SWCNTs/graphene and nanowires. Lee *et al*.^42^ reported CNT/AgNW hybrid nanocomposite by transferring the film from filter membrane to substrate using vacuum suction. The resulted film had sheet resistance of ~27 Ω/sq and 82% transmittance. Haiso *et al*.^35^ reported hybrid films of graphene nanosheet–silver nanowires by dipcoating process with sheet resistance of 71 Ω/sq and 85% light transmittance. Woo *et al*.^43^ prepared a hybrid film by direct mixing of SWCNTs functionalized with quadruple hydrogen bonding (QHB) motifs and aqueous suspension of AgNWs and spray coated onto PET substrate. The resulted film had sheet resistance of ~20 Ω/sq and transparency ~90%. Kholmanov *et al*.^33^ reported hybrid conductive electrodes by solution process using reduced graphene oxide and copper nanowires with sheet resistance of 34 Ω/sq and 80% transparency. Wang *et al*.^44^ demonstrated a solution based method for fabrication of hybrid transparent and flexible electrodes which combines silver nanoparticle networks and thin films of SWCNT network. The resulted hybrid film had sheet resistance ~6 Ω/sq at 83% transmittance. Tokuno *et al*.^41^ reported transparent electrodes using SWCNTs and silver nanowires on plastic films by drop coating the hybrid suspension. Their hybrid electrodes exhibited sheet resistance 29 Ω/sq and 80% transparency. Mao-Xiang Jing *et al*.^45^ used mechanical pressing to prepare hybrid film on PET substrate. The resulted film has sheet resistance ~20 Ω/sq with 84% transparency. The surface roughness after two times of mechanical pressing was ~28 nm. Ju Yeon Woo *et al*.^46^ and Jongsoo Lee *et al*.^47^ reported flexible transparent SWCNT-AgNW hybrid networks on PET substrate by using plasmonic welding to enable electrical stability. Churl Seung Lee *et al*.^48^ fabricated carbon nanotube–silver nanowire network on PET substrate using bar coating method at room temperature. All the above cited papers reported the fabrication of hybrid film on top of rigid or flexible substrate. The novelty of our work is that the hybrid film is embedded in resin and hence the adhesion of the conductive film to the substrate is very good and has good mechanical integrity. Another novelty is that the conducting surface is smooth with surface roughness ~3 nm since the conductive film is embedded inside the resin matrix. Our fabrication method of hybrid conductive film can eliminate the corrosion problem of silver nanowires as the nanaowires are covered by carbon nanotubes. Embedding the hybrid films in resin matrix can (1) improve the bonding between nanowires and nanotubes results in mechanical and electrical stability; (2) the gaps between the nanowires is covered with nanotubes and hence form a smooth, homogeneous and flexible conductive film; (3) improves the mechanical fracture under large bending due to rigidity of nanowires.

We compared the homogeneity of the sheet resistance of our hybrid film containing both SWCNTs and AgNW with the film containing only AgNWs (Fig. 4b). The sheet resistance of AgNW-resin-PET without the SWCNTs (Control) is not consistent and uniform and the sheet resistance varies from 20 Ω/sq to 10^7^ Ω/sq. On many points, the sheet resistance can be as high as ~10^7^ Ω/sq. The high sheet resistance values are obtained when one or more of the four probes are located in the surface region with no AgNWs. If all the four probes contact on the surface region where there is AgNWs, the measured resistance will be low. The SWCNT-AgNW-resin-PET hybrid film shows homogeneous and low sheet resistance of 29 ± 5 Ω/sq all over the surface of the conductive film. The SWCNT network film improves the connectivity of the conductive silver nanowire network and improves the homogeneity of the hybrid film by filling conductive material into the voids in the transferred silver nanowire network. The high conductivity of the SWCNT-AgNW hybrid film on UV cured resin is mainly due to the silver nanowires. We also investigated the endurance of the sheet resistance with bending. SWCNT-AgNW-resin-PET film is flexible under repeated bending stress. After 1000 bending cycles (bending radius of 8 mm), the sheet resistance of the hybrid film remain ~30 Ω/sq (Fig. 4(c)). The sheet resistance of ITO films was reported to increase by almost 2 orders of magnitude after <200 bending cycles^49^. The bending test shows that there is no mechanical degradation of the SWCNT-AgNW-resin-PET film and the sheet resistance after 1000 bending cycles is almost same as the sample without bending. Hence, the fabricated SWCNT-AgNW-resin-PET film has excellent mechanical robustness for adhesion, friction and bending.

**Figure 5:**
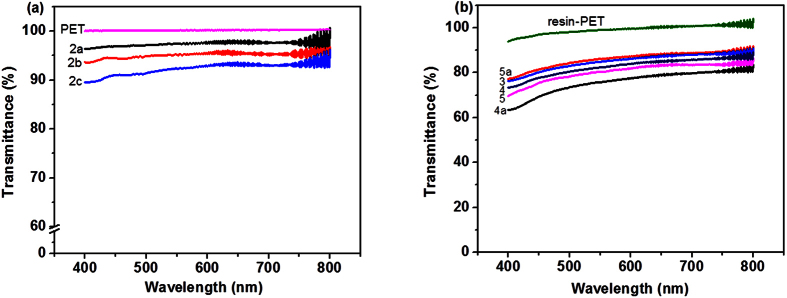
(**a**) Transmittance of SWCNT films on PET with different areal concentration of SWCNTs (Table 1, # 2a-2c); (**b**) Transmittance spectra of SWCNT-AgNW-resin-PET films with different areal concentration of AgNWs and SWCNTs (Table 1, #3–5, 4a and 5a).

We investigated three different areal concentrations of SWCNTs by adding 0.1 ml, 0.2 ml and 0.3 ml of the SWCNT dispersion (0.2 mg/ml) without any AgNWs onto the 47 mm diameter-film disc to prepare the SWCNT network film on PET (Table 1, Formulation 2a-2c). Optical transmittance at 550 nm for 0.1 ml, 0.2 ml and 0.3 ml SWCNTs film are 97%, 95% and 92.5% respectively Table 1 (Fig. 5a). Sheet resistance of these films on PET substrate is high: ~25 kΩ/sq, 12 kΩ/sq and 5 kΩ/sq respectively. These compositions were then added with AgNWs (20 μL/47 mm diameter) dispersion (Table 1, Formulations 3–5 respectively) to form SWCNT-AgNW hybrid films; with fixed AgNW concentration (20 μL/47 mm diameter), and varying SWCNTs volume of 0.1 ml, 0.2 ml, 0.3 ml, it was found that the sheet resistance remains almost the same (~30 Ω/sq) but the transmittance decreases from 85% to 80% as we increase the SWCNTs volume from 0.1 ml to 0.3 ml (Table 1, #3–5; Fig. 5b). This shows that varying the concentration of SWCNTs affect the transmittance but in the non-zero range investigated, it does not affect the sheet resistance. However, our earlier result in Fig. 4b shows that addition of it improves the homogeneity of the sheet resistance.

As we increased the AgNW volume increased to 30 μL (Table 1, #4a), sheet resistance decreased to ~7 Ω/sq but the transmittance also decreased to ~76%. When the AgNW volume decreased to 10 μL (Table 1, #5a), transmittance improved to ~86%, but the sheet resistance increased to 54 Ω/sq. These trends indicate that AgNW networks place a dominant role for the sheet resistance and transparency and SWCNTs network film act as a supporting function to form continuous conductive film. Addition of too much SWCNTs or AgNWs will adversely affect the transmittance of the hybrid film because of the absorption of light by SWCNTs and reflection by AgNWs. We also compared the temperature dependence of the conductivity for our SWCNT-AgNW-resin hybrid film on PET with ITO film on PET (Figure S4). Since PET was used as the carrier substrate, the maximum temperature was chosen was 80 °C. Within the measured temperature range, the conductivity for the hybrid film is stable.

Maximum current through the SWCNT-AgNW hybrid film was studied as a function of AgNW doping and the I-V characteristics of the films with different sheet resistance are shown in Supplementary Figure S5. During the I-V characteristic measurements, the voltage was increased slowly until the sample failed to let current flow through. It was observed that the sample with the lowest sheet resistance could sustain larger maximum current density (around 220 mA) and lower breakdown voltage (around 8 V); the sample with the highest sheet resistance has the lowest maximum current (around 70 mA) and higher breakdown voltage (around 18 V). Hence, the AgNW network affects the breakdown current of our transparent conducting film. Also, films with low sheet resistance have lower breakdown voltage. After the AgNW network breaks down, the film resistance increases and hence the current becomes very small. We also compared the effect of the SWCNT layer on the residual current after breakdown: Figure S6 shows the I-V characteristics of AgNW film and SWCNT-AgNW film embedded in resin. After the breakdown, the current becomes zero for AgNW film but with SWCNT-AgNW hybrid film, a minimum current (around 7 mA) still exists because of the SWCNT layer.

**Figure 6:**
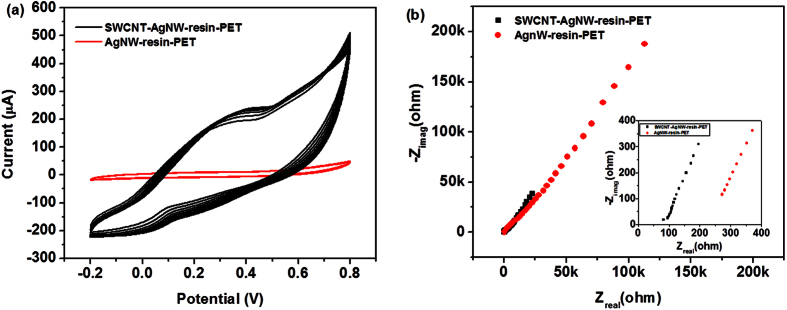
(**a**) CV curves of AgNW-resin-PET film and SWCNT-AgNW-resin-PET film; (**b**) Nyquist plot of AgNW-resin-PET film and SWCNT-AgNW-resin-PET film, inset of (**b**) shows the expanded high frequency region.

Furthermore, anticipating the possible application of the as-prepared conducting films for supercapacitor applications, the electrochemical studies were performed on the transparent film electrode. To analyze the electrode performance, the cyclic voltammetry (CV) and electrochemical impedance spectroscopy (EIS) studies of the hybrid film were carried out. Figure 6(a) shows the cyclic voltammograms of SWCNT-AgNW-resin-PET hybrid film and AgNW-resin-PET film within the potential range of −0.2 to 0.8 V at the scan rate of 10 V/sec. The peak current at 0.8 V for the SWCNT-AgNW-resin-PET hybrid film electrode is found to be ~470 μA, which is about 10 times higher than that of AgNW-resin-PET electrode (~46 μA). The nature of the cyclic voltammograms of the hybrid film is little different compared to the pure AgNW-resin electrode. The hybrid electrode showed pseudo-capacitive nature which is characterized by non-rectangular CV curve of the electrode.

Electrochemical impedance spectroscopy (EIS) study of both the samples was performed with frequency range from 100 KHz to 1 Hz under ac voltage amplitude of 5 mV. The EIS study of the hybrid film is analyzed using Nyquist plot as shown in Fig. 6(b). Inset of Fig. 6(b) shows the expanded high frequency region of the same Nyquist plot. The plot exhibits linear slope for all the measured frequency range. The intercept at Z_real_ along the X-axis known as equivalent series resistance (ESR) is 85 Ω for the SWCNT-AgNW-resin hybrid film, which is much lower than that of 272 Ω for the control film (AgNW-resin). Thus, it can be inferred that the interaction between SWCNTs and AgNWs in the hybrid film shows supercapacitor behavior with improved electrode performance.

**Figure 7:**
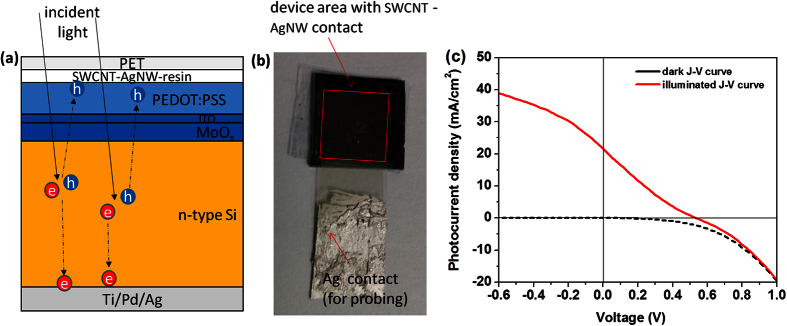
(**a**) Schematic of the structure of solar cell device; (**b**) optical photograph of the solar cell device fabricated using SWCNT-AgNW resin hybrid film as transparent conducting electrode; (**c**) current density–voltage (J-V) plot of the Si heterojunction solar cell with embedded SWCNT-AgNW hybrid film as transparent electrode.

To demonstrate the suitable application of our flat and flexible transparent conducting film, a silicon heterojunction solar cell device with 0.42 cm^2^ contacted area was fabricated using SWCNT-AgNW hybrid film as transparent conducting electrode (see Supporting Section S2.1-Protocols). Figure 7 shows the schematic cross-section of the solar cell device and the current density-voltage characteristics. The current density-voltage (J-V) characteristics were measured by San-EI electric solar simulator system in dark and under standard light illumination of AM1.5G spectrum (full sunlight spectrum) and 100 mW/cm^2^ intensity. The negligible dark current in reverse bias is attributed to the flatness of the SWCNT-AgNW hybrid film. The dark current in reverse bias will be large if there is roughness of the nanowire electrode, which can be extended into the active layer. For the fabricated device, the open circuit voltage is 0.54 V, the short circuit current density is 21.7 mA/cm^2^ and the fill factor is 0.21. This yields a power conversion efficiency of 2.4%. The fill factor can be improved by optimizing the contact between the transparent electrode and the PEDOT:PSS layer.

We also demonstrated the application of our flexible transparent conductive hybrid film to power-up LED. Figure S7 shows the optical photograph of lighting LED connected through SWCNT-AgNW hybrid film. (See also the Supplementary Video).

## Discussion

The major components of our UV resin comprise 1:1 mixture of the urethane diacrylate (EB 270) and ethoxylated bisphenol diacrylate (SR 349) (epoxy-like) and they were chosen for high transparency of the polymeric resin. Different weight ratios of EB 270 urethane acrylate and SR 349 epoxy-like acrylate such as 1:1, 3:1 and 1:3 were attempted for the resin formulation used for making the flat hybrid film. It was found that with 1:1 ratio of EB 270 and SR349, the SWCNT-AgNW hybrid film can be easily peeled off from the original PET substrate. High weight ratio of the epoxy-like SR349 causes the resin film to stick to the PET substrate after UV curing and peeling off the film was quite difficult. The urethane diacrylate increases the flexibility of the resin and also contains the –N(H)- groups that can also bond to AgNW surface^50^,^51^. This bonding may also play a role in transferring the SWCNT-AgNW film onto the UV cured resin. Also, the UV resin formulation must be thoroughly mixed for good homogeneity. Non-uniform mixing may cause uneven curing in different parts of the resin film. The air bubbles must be completely removed by degassing the resin before casting onto the PET substrate. Air bubbles trapped inside the resin formulation can cause damage to the cured resin film. The resin will not cast properly on the PET substrate if the resin formulation has high viscosity. To address these concerns, the sandwich structure consisting of top PET carrier, UV curable resin and the SWCNT-AgNW hybrid film on PET substrate was degassed in a vacuum oven for ~2–3 hrs: at room temperature for about 1–2 hours followed by elevated temperature (~50 °C) for 1–2 hours. Mild heating decreases the viscosity of the resin, which improves its behavior during casting. Also, mild heating can enhance the removal efficiency of trapped air bubbles from the resin formulation, which improves the resin film quality after curing. We used a Meyer rod to coat the resin on PET substrate with SWCNT-AgNW hybrid film. The advantage of this coating process is that it is simple, cost effective and scalable. The UV resin was formulated so that the resin fluid has good rheological flow behavior and wetting properties. The coating dosage must have sufficient material and viscoelasticity to form a uniform, homogeneous and continuous thin layer over the SWCNTs-AgNW. The surface tension of the resin formulation is low since we added a small amount (2%) of silicone polyacrylate (EB350) in order to facilitate proper spreading of resin and avoid discontinuities and de-wetting. Initially the surface of the resin film is corrugated immediate after the coating but it then flattens within a few minutes due to capillary leveling. This coating method is efficient, scalable and produces homogeneous resin films.

We also applied our transfer process to a silver grid pre-adhered on a PET substrate using Cima transparent silver grid film. Our process can successfully transfer silver grid into a UV cured resin to flatten the grid. We then deposit SWCNTs on the flat UV cured resin with embedded silver grid by immersion into a SWCNT suspension in water. The process can also make the conductivity homogeneous (Supporting Information) but the SWCNTs network is not embedded within the UV resin and has less mechanical integrity.

The unique feature of our process is that it produces a flat and smooth surface that is conductive and we have introduced a multi-step process that is solvent-free with little waste generation. Transfer processes have been reported^32^,^36^,^52^ but they typically involve solvent for removal of the sacrificial substrate while we used the lamination-transfer process without any solvent for the clean and green transfer. We applied lamination-transfer which can get good network connectivity. Solvent based membrane removal from SWCNTs is not green and there is also inevitably some contamination from the membrane remaining on the SWCNT film, which degrades the film properties. The advantage of vacuum filtration for preparation of the SWCNTs network film is that it requires only a very small quantity of SWCNT dispersion to achieve the needed areal density of SWCNTs. The AgNW material consumption for the vacuum filtration method is much less than in processes such as spray coating, spin coating *etc*. Vacuum filtration method produces uniform and transparent AgNW conductive films. Cellulose acetate membrane filters were used in our experiments to prepare uniform films. We also tried to use PTFE membrane filter for preparing the AgNW film, but the film was not uniform and there were many streaks observed after transfer of the film to PET substrate.

The effective sheet resistance of the hybrid film can be modeled as a parallel combination of the sheet resistance of the individual components such as silver grid or AgNW network and SWCNT network film. The overall sheet resistance of the hybrid transparent film can be expressed as^53^


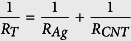


where R_T,_ R_Ag_ and R_CNT_ are the sheet resistances of the hybrid transparent conductor, silver nanowire and SWCNT film respectively. When R_CNT_ ≫ R_Ag_, 1/R_CNT_ will be negligible and R_T_ ~ R_Ag._ Hence the sheet resistance of the hybrid transparent conductive film is largely dominated by the silver nanowire. The SWCNT film is important for filling up the empty spaces bounded by the silver nanowire pattern to form a continuously conducting film. One of the advantages of using SWCNT-AgNW based hybrid film is that the effects of doping can be avoided. Since there is no chemical doping process involved, the fabricated hybrid transparent conductors are free of degradations arising from any kind of doping effect. The carbon nanotube film can act as oxidation resistant sacrificial layer to the silver nanowire and can improve the stability of the SWCNT-AgNW hybrid film^54^.

Our hybrid film has the SWCNT network which is continuous and can act as a bridge to improve the electrical continuity of the silver nanowires network. The network density of the SWCNT film can be adjusted by controlling the concentration of the dispersion solution used in the vacuum filtration step. If only silver nanowires were used to fabricate transparent conductive film by a resin-embedding method, there would be many non-conducting empty spaces, with dimensions of tens of microns, surrounded by the silver nanowire network. Such a film cannot be used efficiently for applications such as flexible OLEDs and photovoltaic devices in which continuous conducting film is needed. In order to achieve low sheet resistance and uniform conductive film, the empty spaces within the silver nanowire network must be covered with another conductive material which has no voids, or at least a much smaller scale void pattern. Such a material would act to bridge the voids in the silver nanowire network. The incorporated material must have high transparency, high conductivity, good conformal coating with silver nanowire network and solution processability^41^. It is reported that carbon nanotubes act as conductive material in hybrid transparent electrodes of graphene and enhance the electric conductivity^55^. Since SWCNTs have high conductivity and transparency, we used carbon nanotube network film to cover the empty spaces bounded by the silver nanowire lines in order to produce a continuous conductive film which is flat but also flexible. These nanotubes are unsorted and much cheaper than high purity sorted metallic tubes and CVD-grown graphene, but with equivalent performance to bridge the AgNWs^44^. The SWCNT dispersion was prepared in 1% SC and the bundles were removed by centrifugation.

A continuously conductive film will be useful for solar cell applications because it decreases the series resistance in the cell, which will increase short-circuit current resulting in higher efficiency^55^. These flat transparent and flexible silver nanowire–carbon nanotube films are desirable for photovoltaic applications because most of the energy in the solar spectrum lies in the visible and near-infrared range, and metals are opaque to visible light. Transmittance through an areally diffuse network of thin metal nanowires will be higher than through a denser network. The optical properties of the hybrid film can be tuned to the requirements of the intended application by controlling the features of silver nanowires such as the areal density, length and diameter.

The CV curve of the hybrid electrode demonstrates the pseudo-capacitive nature of the electrode material. The improved conductivity of the transparent conducting film lead to the easy ion transportation during electrochemical study^56^. It should be mentioned that if in a supercapacitor, only double layer capacitive nature exists, then the shape of the CV curve should be approximately rectangular. However, if there is a contribution of pseudo-capacitive nature of the material, then the shape of the curve will no longer retain its rectangular shape due to the influence of chemical reactions in the presence of electrolyte ions. This observation is also well supported by SWCNT-AgNW-resin hybrid film when it is used as an electrode material; and the shape of the curve is non-rectangular indicating the occurrence of redox reaction in presence of electrolyte ions from Na_2_SO_4_. This can be explained by the incorporation of highly conducting SWCNTs into the hybrid film, where it is postulated that the AgNW in the composite is become ionically very active at higher conductivity in presence of SWCNT, where the redox peak indicates a pseudo-capacitance derived from different oxidation states of Ag^57^,^58^. Also, the excellent current response from the CV curve of the hybrid film indicates the occurrence of efficient faradaic redox reaction at higher electrons and ions transport rate.

Furthermore, the electron and ion transport phenomena in the hybrid film electrode is also analyzed by EIS technique using Nyquist plots. In Nyquist plot there are three main parts–the equivalent series resistance (ESR), interfacial charge transfer resistance (Rct) and the Warburg resistance (Rw)^59^. The intercepts of the curves along the real axis (X-axis) at high frequency indicate the ESR of the material. The ESR originates from - (a) the ionic resistance of the electrolyte within the electrode, (b) intrinsic resistance of the material and (c) the contact resistance at the active material/current collector interface. The lower ESR for the hybrid film is due to good conductivity of the hybrid film sample in presence of SWCNTs compared to the control sample. There is no semicircle at high frequency region (lower left part of the curve) indicating negligible interfacial charge transfer resistance (Rct) during the electrochemical charge-discharge study. Thus, it can be postulated that the interaction between SWCNTs and AgNWs in the hybrid film played a pivotal role for improved electrode performance. Further, the slope of the tails extending in the low frequency region (upper right part of the curve) provides a measure of the electrolyte diffusion resistance which is commonly known as Warburg resistance (Rw). If there is no diffusion resistance, the tails in the low frequency region should be vertical to the real X-axis. Although for the hybrid film, the improvement of the Rw seems little (i.e. for hybrid film, curve at low frequency region is slightly more vertical) compared to the control film, the overall electrochemical characteristics of the hybrid film is superior.

## Conclusion

We have demonstrated the synthesis of a flat transparent conductive SWCNT-AgNW hybrid network embedded in a UV cured resin. The optimized hybrid films have uniform low sheet resistance of 29 ± 5 Ω/sq with high transparency of 85% at λ = 550 nm. Our fabricated hybrid films are mechanically robust; after bending the hybrid film for over 1000 cycles, the sheet resistance of the SWCNT-AgNW-resin-PET film remains consistent and maintain at ~30 Ω/sq. The surface of the film is smooth with rms roughness of 3 nm. Our process involving lamination-transfer printing does not require any organic solvent for removal of the sacrificial substrate and leads to highly consolidated networks embedded within the molded resin with low resistance compared to neat network without any resin cast on the PET substrate. These transparent, conductive, flat and flexible films can be fabricated in large scale at lower cost than the present widely-used transparent conductor, ITO. Furthermore, we also believe that the as-prepared conducting transparent film may also inspire its use as a potential candidate for high energy density flexible-solid-state supercapacitors.

## Experimental Methods

### Chemicals and Materials

P2-SWCNTs used in the experiments were purchased from Carbon Solutions Inc. Sodium cholate (SC), Sodium sulfate and Silver nanowire suspension with average nanowire diameter of 115 nm and length distribution in the range 20–50 μm in isopropyl alcohol (IPA, 0.5 wt% concentration) were purchased from Sigma Aldrich. Silver grids on PET substrate were granted from Cima Nanotech Inc. EB 270 is an aliphatic urethane diacrylate supplied by UCB Chemicals, SR 349 is etoxylated bisphenol A diacrylate supplied by Sartomer Chemicals, dipropylene glycol diacrylate supplied as SR508 by Sartomer Chemicals, trimethylpropane triacrylate (TMPTA) supplied by Aldrich, EB 350 is a silicone polyacrylate supplied by UCB Chemicals and photoinitiator Darocure supplied by Ciba Chemicals.

### Preparation of SWCNT-AgNW film on PET substrate

P2-SWCNT dispersion with a concentration of 0.2 mg/ml was prepared by adding 4 mg SWCNTs in 20 ml of 1% SC solution in water and tip sonicating for 1 hour with 5 second ON, 2 second OFF and 60% power amplitude. The dispersion was then centrifuged at 50,000 RCF for 1 hour. The supernatant was used to prepare a highly diluted SWCNTs suspension (1–3 μg/ml) in DI water. The diluted SWCNT dispersion was then bath sonicated for 15 min. Then, the SWCNT dispersion was filtered through a cellulose acetate membrane filter with 0.2 μm pore size and 47 mm diameter (Step 1, Fig. 1). The SWCNT film from the filter membrane was then transferred onto a PET substrate using the laminator (GBC Inc.) operated at 85 °C (Step 2, Fig. 1).

Separately, very dilute silver nanowire dispersion was prepared by adding ~10–30 μL of the original AgNW suspension in 20 mL of ethanol and bath sonicating for 15 min. The diluted dispersion was then filtered through a cellulose acetate (CA) membrane with 0.2 μm pore size and 47 mm diameter (Step 3, Fig. 1). The silver nanowire film from the filter membrane was then transferred onto the previously prepared SWCNT film on PET substrate using the laminator operated at 85 °C (Step 4, Fig. 1).

### Transfer of SWCNT-AgNW film from PET substrate to UV cured resin

The UV resin formulation that we used for transferring SWCNT-AgNW hybrid film from the PET substrate was a mixture of six components with weight ratio: EB 270 (39%), SR349 (38%), SR 508 (10%), TMPTA (10%), EB 350 (2%) and Darocure (1%). A homogeneous and uniform mixture of the above UV curable resin formulation was prepared and degassed in a vacuum oven. The oven was heated to 40 °C while evacuating to completely remove the air bubbles from the resin mixture. After degassing, the UV formulation was bar-coated (Meyer rod, No. 28) on the SWCNT-AgNW hybrid film on PET substrate. The top surface of the resin film was slowly covered with another PET carrier. The sandwich structure consisting of top PET carrier, UV formulation and the hybrid film on PET substrate was degassed again in a vacuum oven at 50 °C for 2–3 hours to remove the air bubbles trapped within the resin and at the resin PET interface. The sandwich structure (Carrier PET/Resin/SWCNT-AgNW hybrid film) was exposed to UV light for ~20 min. The UV cured resin with carrier PET was then peeled from the original PET substrate. The SWCNT-AgNW hybrid film was completely transferred from the PET substrate to the UV cured resin. Detailed experimental procedure for the preparation of silver grid-SWCNT film on UV cured resin is given in the supporting information.

### Equipment and characterization

The sheet resistance of SWCNT-AgNW hybrid film on UV cured resin was measured with a Keithlink four-point probe station (probe spacing 1.6 mm, probe pin diameter 40 μm). The surface morphology of the hybrid film was performed using a scanning electron microscope (FESEM, JOEL, JSM-6700F) with an accelerating voltage of 5 kV. The surface roughness was measured with a MFP3D AFM microscope under tapping mode. Raman spectroscopy was conducted using a Renishaw Raman microscope with 633 nm laser wavelength. Transmittance spectra were measured using a Varian Carry 5000 UV-vis-NIR spectrophotometer. Cyclic voltametric measurements were performed using an electrochemical workstation (CHI660c, CH instruments) with two electrode configuration in 1 M Na_2_SO_4_ electrolyte. Bending test for the SWCNT-AgNW-resin hybrid film was performed using a glass vial of 16 mm diameter. The hybrid film used to place on the circumference of the glass vial for a required number of cycles and measured the sheet resistance.

## Additional Information

**How to cite this article**: Pillai, S. K. R. *et al*. Totally embedded hybrid thin films of carbon nanotubes and silver nanowires as flat homogenous flexible transparent conductors. *Sci. Rep.* **6**, 38453; doi: 10.1038/srep38453 (2016).

**Publisher’s note:** Springer Nature remains neutral with regard to jurisdictional claims in published maps and institutional affiliations.

## Supplementary Material

Supplementary Video

Supplementary Information

## Figures and Tables

**Table 1:** Sheet resistance and transmittance data for AgNW-resin-PET (#1), SWCNT-PET (#2a-2c) and SWCNT-AgNW-resin-PET films (#3–5, 4a and 5a).

No.	SWCNTs volume (ml)	AgNW volume (μL)	Transmittance (%) at 550 nm	Sheet resistance (Ω/sq)
1	0	20	87	20−10^7^
2a	0.1	0	97	25 × 10^3^
2b	0.2	0	95	12 × 10^3^
2c	0.3	0	92.5	5 × 10^3^
3	0.1	20	85	29 ± 5
4	0.2	20	82.5	29 ± 6
5	0.3	20	80.5	32 ± 3
5a	0.3	10	86	54 ± 5
4a	0.2	30	76	7 ± 1
