# Supplementary material for: Totally embedded hybrid thin films of carbon nanotubes and silver nanowires as flat homogenous flexible transparent conductors
Source: Sci Rep. 2016 Dec 8;6:38453. doi: 10.1038/srep38453 (PMC5144093; doi:10.1038/srep38453)
Supplement: Supplementary Information [file srep38453-s2.doc]

**Supplementary Information**

**Totally embedded hybrid thin films of carbon nanotubes and silver nanowires as flat homogenous flexible transparent conductors**

Suresh Kumar Raman Pillai^1^, Jing Wang^1^, Yilei Wang^1^, Md Moniruzzaman Sk^1^, Ari Bimo Prakoso^2^, Rusli^2^ and Mary B. Chan-Park^1^,*

^1^School of Chemical and Biomedical Engineering

^2^School of Electrical and Electronics Engineering

Nanyang Technological University

62 Nanyang Drive, Singapore 637459

E-mail: MBEChan@ntu.edu.sg

**Supporting Section S1 – Figures**

**Figure S1**: UV-vis-NIR of P2-SWCNT. The optical absorption spectrum of a particular nanotube species is dominated by series of inter-band transitions, at energies denoted E_11_, E_22_, *etc.* associated with van Hove singularities. The absorbance peak in the 600-800 nm range is due to first van Hove electronic transitions of metallic Nanotubes (M_11_) and the peak in the 920-1120 nm range is due to second van Hove electronic transitions of semiconducting Nanotubes (S_22_).

**Figure S2:** Raman spectra of SWCNT-AgNW-resin film with carrier PET (black). Also shows AgNW film on UV resin with carrier PET (red) and PET substrate (blue). Data are normalized with respect to highest peak intensity.

**Figure S3:** sheet resistance with optical transmittance at 550 nm wavelength for different SWCNT-AgNW-resin-PET films.

**Figure S4**: Normalized conductivity with temperature on SWCNT-AgNW resin hybrid film and ITO on PET film.

For the ITO tested in Figure S4 which can be used for mobile phone screens, the general technical specifications are (http://www.ec21.com/product-details/ITO-PET-Film--3849330.html): (i) Sheet resistance ~ 80 ± 10 Ω/sq; and (ii) transparency ≥ 86 %. The SWCNT-AgNW hybrid films that that we fabricated with sheet resistance of 29 Ω/sq and transparency of 85 % is comparable with the commercially available ITO-PET films used in mobile phone screens.

**Figure S5**: I-V characteristics of SWCNT-AgNW hybrid film embedded in resin with different sheet resistance by varying the AgNW doping.

**Figure S6**: I-V characteristics of AgNW film and SWCNT-AgNW hybrid film embedded in resin.

**Figure S7**: Application of SWNT-AgNW hybrid film (circled) as flexible conductor for lighting LED -- Optical photograph of LED circuit connected using the SWCNT-AgNW hybrid film (a) before and (b) after applying DC voltage of 4 V.

Transparent conductive films have wide variety of applications in optoelctronic devices, including flat panel displays, touch screens, solar cells and OLEDs. These conductive films can also be used in smart windows, smart heaters and supercapacitor electrodes. We demonstrate in Figure S7 the application of our flexible transparent conductive hybrid film to power-up LED and presented as supplementary video.

Supplementary Video:

Demonstration of SWCNT-AgNW hybrid film as a flexible transparent conductor to power–up LED

Supporting Section S2 –Protocols

S2.1 Protocol for fabrication of solar cell device using SWCNT-AgNW- resin hybrid film as transparent conducting electrode.

We have fabricated Si heterojunction solar cell device using our SWCNT-AgNW hybrid film as transparent electrode. **Figure 7** shows the schematic cross-section of the solar cell device. N-type Si wafer is cleaned by acetone, IPA, DI water in ultrasonic bath sequentially before immersed in dilute HF solution to remove native oxide. Ti/Pd/Ag layer with 20/20/200 nm thickness was deposited at the backside of n-type Si substate with e-beam evaporation. A 15 nm layer of molybdenum oxide (MoOx) is evaporated on top of Si substrate as electron blocking layer, forming p-n junction with n-Si. Thin 5 nm ITO is evaporated on top of the MoOx layer to protect it from water and moisture in subsequent process. All evaporation is done at mid 10^-6^ mbar pressure range using the same e-beam evaporation machine. A water based solution of PEDOT:PSS from Clevious PH1000, was mixed with 5% DMSO and 0.25% fluorosurfactant to improve its conductivity and its wettability respectively. The solution was spin coated on top of MoOx layer at 3000 rpm for 20 s, and annealed at hot plate in ambient air at 105 °C for 5 min. The PEDOT:PSS act as anti reflection coating with thickness ≈ 70 nm and also hole transport layer. Top contact of the solar cell was made by pressing the SWCNT-AgNW transparent hybrid film on top of the PEDOT:PSS layer.

**S2.2 Preparation of SWCNT-Silver grid film on UV cured resin:**

We used silver grid (from Cima Nanotech Inc.) to fabricate our transferred silver grid film. UV curable resin materials were used for transferring the silver grid from original Cima film. The UV formulation was thoroughly mixed using a mechanical stirrer and degassed in vacuum oven. The oven was heated to ~ 40 °C during evacuation to remove the remaining air bubbles. After degassing, the above UV formulation was coated on a (3-Aminopropyl) triethoxysilane (APTES) treated Cima film (silver grid with PET) using a bar coater (Meyer rod, No. 28). The resin was dropped onto the Cima film and the Meyer rod was pulled over the resin to form a thin resin film on the Cima film. The surface of the resin film was slowly covered with another PET carrier. The sandwich structure consisting of top PET carrier, UV resin and original Cima film was degassed again in vacuum oven to remove air bubbles trapped within the resin and at the resin PET interface. The sandwich structure (Carrier PET/Resin/Cima film) was exposed to UV light for ~ 20 min. The cured resin with carrier PET was then peeled off. The silver grid was completely transferred onto the cured resin with carrier PET. SWCNT dispersion was prepared by dispersing ~ 4mg P2-SWCNT in 20 ml of 1 % SC (Sodium Cholate) solution in water and tip sonicating for 1 hour with 5 second ON, 2 second OFF and 60% Amplitude. The dispersion was then centrifuged at 50,000 RCF for 1 hour and the supernatant was collected. SWCNT film was deposited on the transferred silver grid on UV cured resin by dip coating or immersion process.

**Figure S8**: Schematic diagram of the fabrication procedure for flat Ag grid-SWCNT hybrid transparent conductive film on UV cured resin.

**Figure S8** shows a schematic of the fabrication procedure for hybrid film using transferred silver grid on UV cured resin and carbon nanotube network film by solution based coating process. The sandwich structure consist of top PET carrier, UV curable resin and Cima film was degassed in a vacuum oven for ~ 2-3 hours, heated to ~ 50°C. UV exposure of the sample was done using a 365 nm UV light source. The completely cured sandwich structure was removed from the UV chamber and the cured resin, carrying the silver grid, was peeled from the Cima film substrate.

**Figure S9**: Visual appearance of 3 samples of the silver grid transferred on UV cured resin with carrier PET.

**Figure S9** shows visual images of three samples of transferred silver grid on UV cured resin. The size of the samples is ~ 6 cm x 4 cm. The film is clean and the silver grid is almost completely embedded within the resin film due to the transfer process. The objective of transferring the silver grid on resin is to produce a flattened conductive film. The electrical continuity of the transferred silver grid on resin was measured by a multimeter with two probes in contact with two extreme edges of the sample. The completely cured sample is flexible and transparent.

**Figure S10:** AFM images (i, ii, and iii corresponds to height, amplitude and phase images respectively) of the transferred silver grid on the UV cured resin. (iv), Microscopic image of the transferred grid on resin where AFM scan has done.

The surface of the resin with transferred silver grid was characterized with AFM. Images at different positions on the film were taken. **Figure S10** shows the AFM images (height, phase and amplitude) of the grid line on the resin surface. The height trace of the images shows that mostly the transferred grids on the resin surface are flat with height ~ 100 nm. Some protrusion of silver on the edges of the grid lines with height of <200 nm is observed. This may be due to the difference in material properties of UV resin and silver. The phase and amplitude images support the flattening of the transferred silver grid on resin film. The AFM results show that most of the silver particles might be embedded within the UV cured resin after the transfer process. The silver grid on the original Cima film substrate has height of ~ 3-5 µm.

By transferring the silver grid on to UV cured resin, the surface relief of the silver grid that remains exposed is reduced to <200 nm from an initial relief (on the Cima film) of ~3-5 microns. Commercially available silver grid on PET (Cima NanoTech) is prepared by large scale solution based self assembly of silver nanoparticles. There are many non-conductive voids surrounded by the silver grid network. The size of these empty spaces varies from 100 µm to 500 µm. The silver grid pattern is unchanged by transferring onto UV cured resin. Usually, films with this pattern of many empty spaces cannot be used for applications in which continuous conducting film is needed. In order to achieve low sheet resistance and uniform conductive film, the empty spaces within the silver grid network must be covered with another conductive material which has no voids, or at least a much smaller scale void pattern. This material will act to bridge the voids in the silver grid network.  Since SWCNTs have high conductivity and transparency, we used carbon nanotube network film to cover the empty spaces bounded by the silver grid lines in order to produce a continuous conductive film which is flexible and flattened.

To obtain good adhesion of SWCNTs, the transferred silver grid with UV cured resin was treated with O_2_/Ar plasma for 2 min followed by APTES treatment. Plasma treatment makes the surface hydrophilic by increasing the density of hydroxyl groups, which is useful for introducing amine groups during the APTES treatment.^1^ The cured resin film with grid was placed in a solution of 1 vol % of APTES in Isopropyl alcohol (IPA) for 20 min, then rinsed with IPA to remove excess APTES and then dried in an oven at 70 °C. SWCNTs have good affinity to amino groups so that SWCNT deposition is improved by APTES treatment. Proper plasma and APTES treatment is necessary for uniform deposition of SWCNT film onto the silver grid/resin surface. SWCNT film was deposited on the amino functionalized UV cured resin with silver grid by dip coating or immersion process.

1. **(B)**

**Figure S11**: AFM images of SWCNT network film in the empty space between the silver grid on resin substrate (A) immersion process (B) dip coating process.

**Figure S11** shows an AFM image of the SWCNT network film in an empty space of a silver grid embedded in resin. The SWCNT network is quite dense with multiple SWCNTs passing through every square micron of the film surface. The continuous SWCNT network film acts as a bridge for the silver grid lines. The network density of the SWCNT film can be adjusted by controlling the number of dip-coating cycles, the concentration of SWCNTs in the dispersion solution, *etc*. The flatness of the silver grid embedded in resin makes it possible to deposit a very flat and thin SWCNT network via dip-coating, resulting in a very flat continuous conductive film. Immersion process of depositing SWCNT film is an alternative method to produce a uniform SWCNT film on cured resin with transferred silver grid. These two methods permit re-use of the SWCNT dispersion, which is less costly than methods such as spray-coating. These methods are also scalable and safe compared to spray-coating method that generates aerosolized SWCNTs.

**Figure S12**: (a) Visual appearance of transferred silver grid on UV cured resin, (b) Visual appearance of the SWCNT-Ag grid hybrid film on UV cured resin. The SWCNT film was deposited by immersion process. (c) Microscopic image of the hybrid film on UV cured resin marked in red square.

**Figure S12** shows the visual appearance of resin-embedded silver grid and hybrid film of SWCNTs on resin-embedded silver grid. Figure S12(c) is a microscopic image of the marked area of the hybrid film on Figure S12 (b). The scale bar is 200 µm. The SWCNT film shown in Figure S12 (b) was deposited by immersion process. The transferred silver grid is clearly visible in the microscope image. No breakage of the silver lines is observed due to the transfer process. The silver grid from the original Cima film is completely transferred to the UV cured resin with most of the silver particles embedded within the resin film, resulting in a nearly flat transparent conductive film. The UV curable resin was formulated to yield a well cured (*i.e*., thoroughly cross-linked) film that is both flexible and strong enough to lift the silver grid completely off the original Cima film.

**Figure S13**: Transmittance of Ag grid-SWCNT hybrid film on UV cured resin (blue) and Silver grid (without SWCNT film) on UV cured resin (red).

**Figure S13** shows the transmittance curves of resin-embedded silver grid and hybrid film of SWCNTs on resin-embedded silver grid. The SWCNT film was deposited by immersion process. Optical transmittance of the resin-embedded silver grid is ~ 81 % at 550 nm. The transmittance is consistent and uniform throughout the scanned wavelength region (400-800 nm). The optical properties of the hybrid film can be improved by controlling the silver grid properties such as the width and thickness of the grid lines and the fraction of the film area covered with metal. Since we used standard silver grid film from Cima NanoTech Inc. for our transfer technique, we couldn’t vary these parameters. The transmittance of the Ag grid-SWCNT hybrid film is ~71%. Though the transmittance of the hybrid film reduced, the conductivity of the hybrid film is significantly improved as shown in Figure S14. The sheet resistance was measured by Keithlink four point probe station.

**Figure S14**: Sheet resistance of resin-embedded Ag grid (black squares), SWCNT film (red circles) and SWCNT-Ag grid hybrid film (blue triangles).

**Figure S14** shows sheet resistance measurements for SWCNT film, resin-embedded Ag grid and transparent SWCNT-Ag grid hybrid film. The resin-embedded silver grid behaves like a non-conductive surface. We measured the sheet resistance using a four point probe measurement system. High sheet resistance values are obtained when one or more of the four probes contact the film in an empty space within the silver grid. The measured sheet resistance is very low only when all four probes contact the silver grid. The Ag grid-SWCNT hybrid film shows homogeneous and low sheet resistance (14±2.3 Ω/) over the entire surface of the conductive film. The sheet resistance of the SWCNT film itself is also quite high (7.8 kΩ/). The high conductivity of the SWCNT-Ag grid hybrid film on UV cured resin is mainly due to the silver grid; the SWCNT film puts the intra-grid void spaces into electrical contact with the low-resistance silver grid. The SWCNT network film helps to bridge the conductive silver grid network and improves the homogeneity of the hybrid film by electrically bridging the voids in the transferred silver grid network.

**References:**

1. Roberts, M.E., LeMieux, M.C., Sokolov, A.N. & Bao, Z. Self-sorted nanotube networks on polymer dielectrics for low-voltage thin-film transistors. *Nano Letters* **9**, 2526-2531 (2009).
